# Dietary Verbascoside Influences Gut Morphology and the Expression of α-Transducin and α-Gustducin in the Small Intestine of Weaned Piglets Exposed to n-6 Polyunsaturated Fatty Acids-Induced Oxidative Stress

**DOI:** 10.3390/ani9010020

**Published:** 2019-01-10

**Authors:** Raffaella Rossi, Carlo Corino, Silvia Modina, Alessia Di Giancamillo

**Affiliations:** Department of Health, Animal Science and Food Safety, University of Milan, Via Celoria 10, 20133 Milan, Italy; raffaella.rossi@unimi.it (R.R.); carlo.corino@unimi.it (C.C.); silvia.modina@unimi.it (S.M.)

**Keywords:** piglets, verbascoside, α-transducin, α-gustducin, gut

## Abstract

**Simple Summary:**

In recent years, feed interventions with natural antioxidants have grown because oxidative stress is linked to the development of several diseases in pigs. Weaning is one of the most stressful events in pig breeding, inducing physiological, environmental, and social changes. These cause intestinal disorder, inflammation and oxidative stress that result in reduced pig health and growth. Previous data reported that dietary supplementation with a natural extract containing verbascoside positively affects piglets’ health and welfare by reducing oxidative stress parameters. This study shows that verbascoside protects the gastrointestinal tract from oxidative stress and may have an appetite-stimulatory effect by modulating gastrointestinal taste receptors’ related protein expression.

**Abstract:**

Reducing oxidative stress is an important goal in post-weaning piglets; previous studies have reported that verbascoside decreases oxidative stress in piglets. The effect of verbascoside on gut morphology and α-transducin and α-gustducin expression in weaned piglets fed high dosages of sunflower oil, inducing oxidative stress, was evaluated. A diet with 9% sunflower oil (T1), the same diet supplemented with 5 mg of verbascoside/kg feed (T2) and a diet containing starch (control—CTR) were employed. Histology, histometry, histochemistry, immunofluorescence and Western blot analyses were performed on the piglets’ small intestine. In the T1 group, apical erosion was observed and villi height was lower than in other groups. The mucin profile was acidic in goblet cells of both the T1 and T2 groups. However, it was both neutral and acidic in the CTR group. Dietary treatments did not affect α-gustducin expression. Otherwise, the expression of α-transducin in the duodenum was lower (*p* < 0.01) in the T1 groups than in the other groups. The colocalization of α-transducin with chromogranin A and ghrelin revealed that the endocrine cells were immunopositive for both ghrelin and α-transducin. Overall, these results provide new insights into gut sensory perception in piglets and contribute to understanding how feed ingredients such as fat and polyphenols may be involved in gustatory signal transduction.

## 1. Introduction

Verbascoside is a phenylethanoid glycoside, extensively spread in the plant kingdom, that possesses several biological properties, including anti-inflammatory, antineoplastic, wound-healing, neuroprotective and antimicrobic proprieties [1]. Verbascoside also has powerful antioxidant activity, as indicated in many experimental models [2]. Oxidative damage of tissues and cellular components is an important cause of diseases, related to the welfare and productivity of livestock [3,4]. In piglets in particular, evidence suggests that weaning disrupts the physiologic hepatic equilibrium of oxidants and antioxidants, and leads to oxidative stress [5]. Our previous research demonstrated that dietary verbascoside contrasts oxidative stress mechanisms in post-weaned piglets [6] by inducing a reduction of local stress biomarkers on duodenum endocrine cells [7]. Diet plays an important role in gastrointestinal (GI) metabolism by stimulating the GI chemosensory systems, characterized by endocrine cells lying in the mucosa and by nerve fibers, especially of a vagal nature [8]. A wide range of endocrine and/or paracrine mediators can be released by endocrine cells in response to dietary nutrients [9]; these hormones, in addition to their direct activity, act through specific receptors by activating the control of important functions such as caloric intake and energetic homeostasis of the organism. Moreover, they modulate taste perception. Taste receptors (TR) belong to the family of G-related receptors, a system of the GI tract [10]; this system discriminates the basic taste classes (salty, sour, sweet, umami and bitter) and can associate the feed composition to a feedback pathway that monitors appetite, gut motility and secretion through the gut–brain axis [11]. Therefore, taste perception can affect feed intake and, consequently, performances in livestock [12]. For piglets, weaning is a stressful phase characterized by reduced feed intake that is caused by several factors, for example, the change in composition and physical form of feed. Feed intake is usually reduced at the beginning of weaning and the pig may develop anorexia of variable duration. Underfeeding induces mucosa atrophy in the small intestine and modifies the number of α-gustducin (α-gust) immunoreactive cells in the duodenum and jejunum [13]. Moreover, a recent study indicates that in pigs, high-fat diets induce changes in the taste-related signaling proteins, α-gust and α-transducin (α-tran), along the GI tract [14], suggesting a selective effect of nutrients on taste receptors.

To the authors’ knowledge, no study reported the effects of oxidative stress caused by a high intake of sunflower oil and/or verbascoside on α-gust and α-tran expression in the small intestine of weaned piglets. In a previous study, we demonstrated that high supplementation of sunflower oil induces oxidative stress, decreasing blood and red blood cells’ antiradical activity and plasma catalase activity; moreover, we also showed that dietary verbascoside is able to modulate oxidative damage in piglets’ liver [15].

Since, during the weaning phase, oxidative stress negatively affect piglets’ health, as previously observed [15], and feed ingredients could modulate taste signaling molecules and receptors in the GI tract and feed intake [13,14], our study aimed to test whether dietary high sunflower oil intake with or without verbascoside could also affect GI tract morphology and the expression of α-gust and α-tran immunoreactive cells of weaned piglets.

## 2. Materials and Methods 

Procedures involving animals were carried out in accordance with the European Communities Council Directive (86/609/EEC, 1986) and were approved by the Italian Ministry of Health (Law No. 116/92). 

### 2.1. Animals and Experimental Design 

The experimental design and the composition and chemical analysis of the experimental diets have already been described [15]; the laboratory procedures performed in the present study have been conducted on the same animals enrolled in our previous work [15]. Briefly, 24 weaned female Hypor piglets were housed in the Experimental Zootechnical Center of the University of Milan (Lodi, Italy) and were reared in 24 pens (1 piglet/pen) in an environmentally controlled room. After 7 d of adaptation (10.9 ± 0.1 kg average BW), the piglets were assigned to one of three dietary treatments. The first group was fed a diet with 9% sunflower oil (T1; n-6 polyunsaturated fatty acids high dosage and oxidative stress inducing). The second group was fed the T1 diet supplemented with an extract of Verbenaceae (Lippia spp.) leaves titrated in verbascoside to obtain 5 mg verbascoside/kg feed (T2; n-6 polyunsaturated fatty acids high dosage plus antioxidant). The supplement was microencapsulated within a protective matrix of hydrogenated vegetable lipids using spray cooling technology (Sintal Zootecnica, Isola Vicentina, Vicenza, Italy). The third group was fed a control diet (CTR), in which an isoenergetic replacement of oil by starch was added. 

To highlight the effects of the dietary oxidative stress conditions, all the rations had the same protein and energy content by the adoption of an appropriate ratio between the T1 and T2 groups and the CTR group (1:1.13). The animals were restrictively fed 2.7 times the maintenance requirements for energy (NRC, 1998). The trial lasted 30 days and the growth parameters were recorded. The non-fasting animals were slaughtered at an average live weight of 22 ± 0.3 kg.

### 2.2. Tissue Sampling

The piglets were slaughtered by approved procedures (Italian Ministry of Health; DL 333/1998). For micro-anatomical (histology, histochemistry and immunofluorescence) analyses and Western blot, 1 cm^3^ samples of proximal duodenum (5 cm after the pylorus), proximal jejunum (5 cm after the beginning of the duodenojejunal flexure) and ileum (at the level of the ileocecal fold) were immediately excised from each pig (*n* = 24). For micro-anatomical analyses, the samples were promptly fixed in 4% para-formaldehyde in 0.01 M phosphate-buffered saline (PBS) pH 7.4 for no longer than 24 h at 4 °C, dehydrated in a graded series of ethanol, cleared with xylene, and embedded in paraffin. The samples for Western blot analyses were vacuum-packed and stored at −80 °C until pending analyses. 

### 2.3. Histology and Histometry

Sample microtome sections measuring 4 µm in thickness were stained with hematoxylin eosin (HE) sequential stain to ascertain the structural details. Histometric evaluations such as the height of intestinal villi (V) (five villi measured per section), the depth of intestinal crypts (C) (five crypts measured per section), and the ratio of villi and crypts values (V:C ratio) were performed on HE stained sections and calculated by image analysis software (Image pro Plus 6.3 Media Cybernetics Inc., Silver Springs, FL, USA). 

### 2.4. Histochemistry 

The mucin profile of the duodenum, jejunum, and ileum was determined by staining sections with the Alcian blue 8GX, pH 2.5-periodic acid Schiff combination (AB-PAS), which reveals neutral (PAS-reactive) and acidic (AB-reactive) glycoconjugates [16]. These histochemical reactions selectively evidence both the intestinal mucus cells and the adherent mucus gel, which is synthesized by the mucus cells and lies upon the mucosal epithelial layer. The thickness of the adherent mucus gel, determined as the distance from the outermost layer of the adherent mucus gel itself to the luminal surface of the mucosal epithelial layer, was measured at 100× magnification in five randomly selected points in each AB-PAS stained-section according to Di Giancamillo et al. [17]. 

### 2.5. Tissue Homogenization and Western Blot Analysis

The intestine samples were homogenized in 2 mL of ice-cold lysis buffer (50 mM Tris HCl, pH 7.5, 0.2% Triton X-100, and protease inhibitor mixture) per 200 mg of tissue using an Ultra-Turrax homogenizer (IKA-Werke, Staufen, Germany). For each sample, the homogenate was then centrifuged at 20,000× *g* for 20 min at 4 °C to remove all insoluble material. The supernatant was collected, and total protein content was measured using a commercial protein quantification kit (Pierce, Rockford, IL, USA). The optical densities were read at 562 nm against a calibration curve using Bovine Serum Albumin (BSA Sigma-Aldrich, St. Louis, MO, USA) with a working range of 50 to 800 μg/mL. The total proteins (35 μg) were resolved by 10% Sodium Dodecyl Sulphate - PolyAcrylamide Gel Electrophoresis (SDS-PAGE) and electrotransferred onto nitrocellulose membranes. After blocking, the membranes were incubated overnight at 4 °C with primary antibodies (dilution 1:1000; α-transducin sc-390, and α-gustducin, sc-395, Santa Cruz Biotechnology, Dallas, TX, USA) and, subsequently, with a horseradish peroxidase-conjugated secondary antibody (1:5000) at room temperature for 45 min. Immunoreactivity was detected by chemiluminescence autoradiography according to the manufacturer’s instructions (Biorad, Milan, Italy), and the images were scanned. The optical intensities of the protein bands of interest were determined densitometrically using Scion Image software (Scion Corporation, Frederick, MA, USA). The values were normalized to glyceraldehyde 3-phosphate dehydrogenase levels (clone GAPDH-71.1; Sigma-Aldrich).

### 2.6. Double Immunofluorescence 

Western blot analysis only revealed verbascoside influence on α-tran expression in the duodenum (see below), thus we opted to characterize the cellular typology involved using a double immunofluorescence procedure. De-waxed and rehydrated paraffin sections were incubated with the first-step primary antibody, 1:10 goat anti-mouse α-tran, for 24 h at 18–20 °C, then washed in Tris-buffered saline solution (TBS 0.05 M Tris/HCl, 0.15 M NaCl pH = 7.6), and subsequently treated with the avidin–biotin blocking kit solution (Vector Laboratories Inc., Burlingame, CA, USA). The sections were then washed in TBS for 10 min and incubated with a 10 µg/ml solution of goat biotinylated anti-rabbit IgG (Vector Laboratories Inc.) in TBS for 1 h at 18–20 °C. After rinsing twice in TBS, the sections were treated with 10 µg/ml fluorescein–avidin D (Vector Laboratories Inc, Burlingame, CA, USA), in NaHCO3, 0.1 M, pH 8.5, 0.15 M NaCl for 1 h at 18–20 °C and then washed in TBS and incubated with rabbit IgG (Vector Laboratories Inc.) for 2h to inhibit the binding of the second primary antiserum to the goat anti-rabbit IgG used in the first sequence. For the second step of the double immunofluorescence procedure, the slides were incubated with anti-chromogranin or anti-ghrelin (dilution 1:100; Chromogranin A MON 9014, Cosmobio, Tokyo, Japan; or ghrelin sc-10368, Santa Cruz Biotechnology Santa Cruz, CA, USA), respectively). The sections were then rinsed in TBS for 10 min and incubated with 10 µg mL^−1^ goat biotinylated anti-rabbit IgG (Vector Labs, Burlingame, CA, USA) for 1 h at 18–20 °C, washed twice in TBS, and incubated with 10 µg mL^−1^ rhodamine–avidin D (Vector Laboratories Inc, Burlingame, CA, USA), in NaHCO_3_, 0.1 M, pH 8.5, and with 0.15 M NaCl for 1 h at 18–20 °C. Finally, the sections were mounted with Vectashield Mounting Medium (Vector Laboratories Inc, Burlingame, CA, USA) and observed using a Confocal Laser Scanning Microscope (FluoView FV300, Olympus, Milan, Italy). The immunofluoropositive structures were excited using Argon/Helio-Neon-Green lasers, with excitation and barrier filters set for fluorescein and rhodamine. Images containing the superimposition of fluorescence were obtained by sequentially acquiring the image slice of each laser excitation or channel. Immunoreactive cells were counted by image analysis software in 10 fields (each field represented a tissue section area of about 0.036 mm^2^) [7]. The results were presented as a percentage of α-tran/cromoA or ghre-IR cells. The observer was not aware of the origin of the sections.

### 2.7. Statistical Analyses 

All analyses were performed with SAS software (version 9.3; SAS Inst. Inc., Cary, NC, USA), and a value of *p* < 0.05 was used to indicate statistical significance. One-way ANOVA was used to determine the effects of dietary treatments on feed intake and Western blot analyses. Histometric analyses (villi height, crypts depth, V:C ratio, mucus layer thickness and cell counts) were analyzed by ANOVA using the PROC MIXED procedure of the SAS package. The mixed model included the fixed effects of treatment and the random effect of the piglet. The individual piglet values were considered the experimental unit of all response variables. The data were presented as least squared means ± SEM.

## 3. Results

### 3.1. Growth Performances

No significant differences in final weight and average daily gain were observed and the gain to feed ratio was higher in piglets fed oil-supplemented diets than in the controls [15]. As expected, feed intake was lower (*p* < 0.001) in animals fed 9% sunflower oil than in the controls (589 ± 14 g/d T1 vs. 600 ± 7 g/d T2 vs. 661 ± 8 g/d CTR) due to the restrictive regimen in order to have the same energy and protein intake.

### 3.2. Intestinal Histology and Histometry

**Figure 1:**
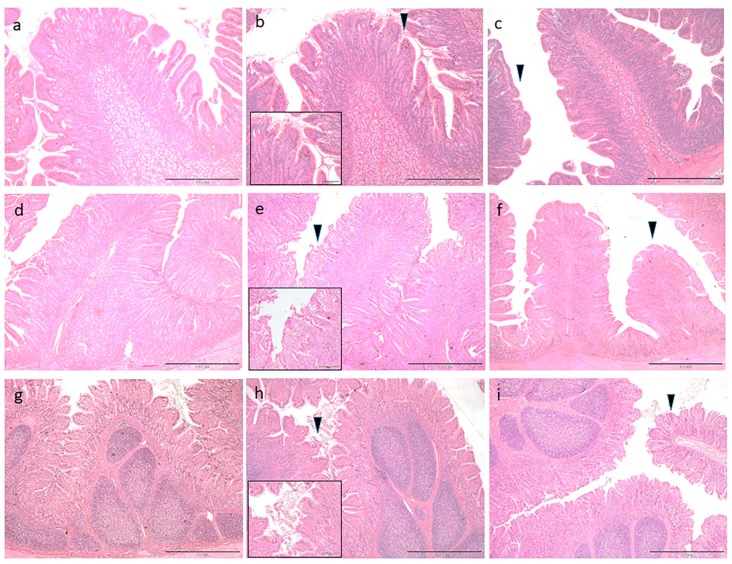
Histological findings of sections of the small intestine (hematoxylin eosin staining) in each group: (**a**) duodenum, control group (CTR); (**b**) duodenum, diet with 9% sunflower oil (T1); (**c**) duodenum, T1 supplemented with 5 mg of verbascoside/kg feed (T2); (**d**) jejunum, CTR; (**e**) jejunum, T1; (**f**) jejunum, T2, (**g**) ileum, CTR; (**h**) ileum, T1; and (**i**) ileum, T2. The arrowheads indicate erosion of the tips of the villi. The insert is an enlargement of the arrow zone, showing epithelial cells debris and the irregular profile of the villi.

Histological evaluation of the small intestine is presented in Figure 1. Piglets fed the CTR diet revealed a normal structure of the duodenum, jejunum and ileum (Figure 1a,d,g, respectively). On the contrary, dietary supplementation with sunflower oil (T1) caused detrimental effects on the mucosa at the top of the villi (Figure 1b,e,h, arrowheads). Briefly, the tips of the villi appeared ragged and irregular and epithelial cells debris from the disrupted villi was detected in the intestinal lumen (Figure 1b, insert as representative image). Finally, the verbascoside dietary supplement (T2) protected the intestinal villi from oxidative damage, so the morphology revealed to be adequately preserved in all the tracts (Figure 1c,f,i, arrowheads). 

The histometric analyses are reported in Table 1 The villi height was significantly higher in the CTR and T2 groups vs. T1 group in the duodenum and jejunum (*p* < 0.01). However, in the ileum, there were significant differences in CTR vs. T1 and T2 groups (*p* < 0.01). 

The crypts depth in the duodenum and jejunum was similar (*p* > 0.05) in all groups. However, the crypts depth in the ileum of CTR animals was significantly deeper when compared to T1 animals (*p* < 0.05). The crypts depth in the T2 group did not differ; neither did it differ in the CTR and T1 groups. The V:C ratio revealed the same trend in the duodenum and jejunum: CTR and T2 groups vs. T1 were significantly higher (*p* < 0.01). However, in the ileum, there were differences between all groups (CTR vs. T1 vs. T2, *p* < 0.01 all comparisons).

### 3.3. Mucins Profile

**Figure 2:**
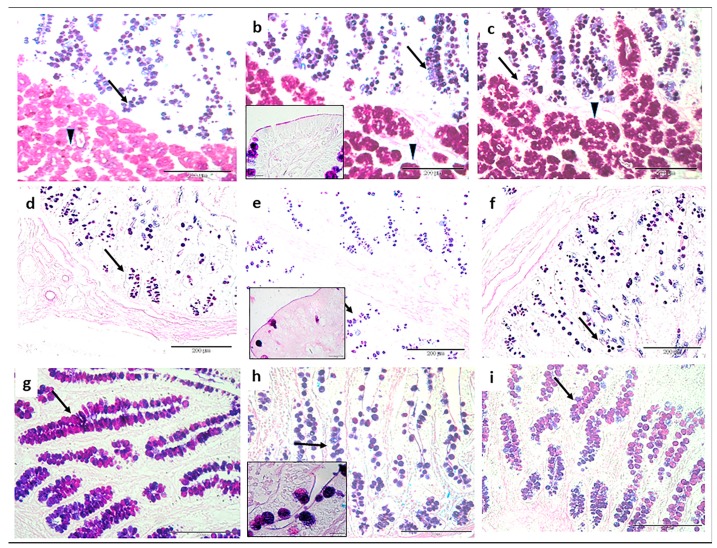
Histochemical findings of sections of the small intestine (Alcian blue 8GX, pH 2.5-periodic acid-Schiff combination (AB-PAS) staining) in each group: (**a**) duodenum, CTR; (**b**) duodenum, T1; (**c**) duodenum, T2; (**d**) jejunum, CTR; (**e**) jejunum, T1; (**f**) jejunum, T2, (**g**) ileum, CTR; (**h**) ileum, T1; and (**i**) ileum, T2. The arrows indicate intestinal goblet cells; the arrowheads indicate Brunner’s glands; the blue color indicates acidic mucins; and the pink color indicates neutral mucins. The inserts (**b**,**e**,**h**) are representative images at a high magnification (100 ×) of the mucosal layer.

**Figure 3:**
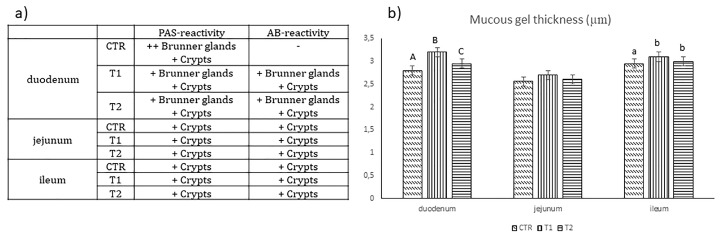
(**a**). Qualitative evaluation of the neutral and/or acidic glyconjugates; (**b**) the histometrical findings of the mucus gel thickness in the duodenum, jejunum and ileum of CTR, T1 and T2 animals (CTR—control diet; T1—sunflower oil supplemented diet; T2—sunflower oil supplemented diet plus verbascoside). The values are shown with different superscripts (a, b) for *p* < 0.05 and (A, B) for *p* < 0.01.

Histochemistry showed the presence of both acidic mucins (blue color) and neutral mucins (pink color) in all the tracts of the small intestine (Figure 2 and Figure 3a). Acidic mucins predominated in all sites; neutral mucins occurred mainly at the bases of the crypts (Figure 2a–c, duodenum sections of CTR, T1 and T2 groups; Figure 2d–f, jejunum sections of CTR, T1 and T2 groups; Figure 2g–i ileum sections of CTR, T1 and T2 groups, respectively; arrows). Duodenal Brunner’s glands seemed predominately to be periodic acid-Schiff (PAS)-reactive in CTR animals, while they seemed more acidic in T1 and T2 groups (Figure 2a–c, arrowheads). The adherent mucus gel AB-PAS staining showed a mixed reactivity. In the duodenum, the T1 layer was thicker than in CTR and T2 groups (*p* < 0.01, Figure 3b). No significant differences were observed in the jejunum. However, in the ileum, the CTR layer was significantly lower compared to T1 and T2 groups (*p* < 0.05). 

### 3.4. Expression of Taste-Related Proteins

**Figure 4:**
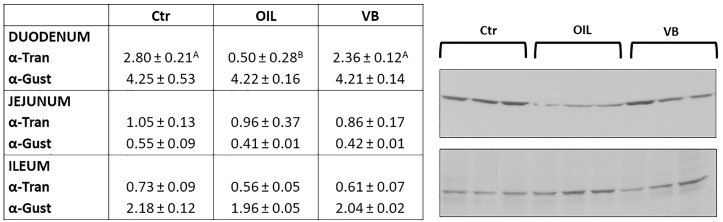
Western blot findings of α-tran and α-gust in the small intestine of CTR, T1 and T2 animals. The values are shown with different superscripts (A, B) for *p* < 0.01.

The antibodies used recognized molecules with two specific molecular weights: α-tran- of a molecular weight ~45 kDa and α-gust of a molecular weight ~40 kDa. A comparison of the relative densities using the anti-α-tran revealed differences among the groups in the duodenum: CTR and T2 groups showed a higher expression than the T1 group (*p* < 0.01), while no differences were detected in the jejunum or ileum. No differences were detected among groups in all the intestinal tracts for α-gust (Figure 4).

### 3.5. Colocalization of α-tran with Chromogranin A or Ghrelin (in the Duodenum)

**Figure 5:**
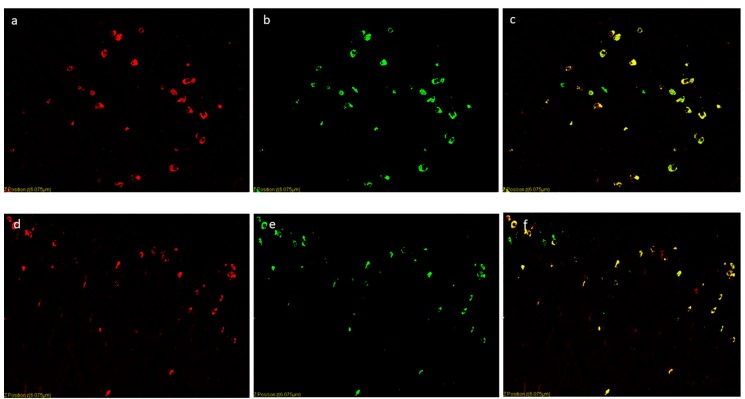
Immunofluorescence findings of the enteroendocrine cells of the proximal duodenum: representative images. Double staining indicates the cytoplasmic localization of chromogranin A, or ghrelin and α-tran. (**a**) CTR, the red color is α-tran; (**b**) CTR, the green color is chromogranin A; (**c**) CTR, the red color is α-tran, the green color is chromogranin A and the yellow color is colocalization. (**d**) T2, the red color is α-tran; (**e**) T2, the green is color ghrelin; (**c**) T2, the red color is α-tran, the green is color ghrelin and yellow color is colocalization.

**Figure 6:**
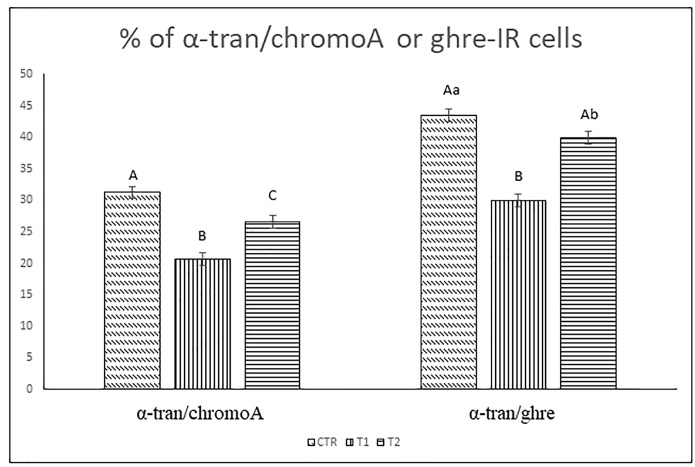
Percentage of α-tran/chromoA or ghre-IR cells. The values are shown with different superscripts (a, b) for *p* < 0.05 and (A, B) for *p* < 0.01.

α-tran immunoreactive cells (Figure 5a,d respectively, red color) and chromogranin A or ghrelin immunopositive cells (Figure 5b,e respectively, green color) were detected and co-expressions with both chromogranin A or ghrelin were observed in endocrine cells (Figure 5c,f respectively, yellow color). Cellular counts revealed that the percentage of α-tran/chromoA-IR cells was significantly higher in CTR vs. T1 vs. T2 groups (*p* < 0.01, all comparisons Figure 6); moreover, α-tran/ghre IR-cells were significantly different in CTR/T2 vs. T1 (*p* < 0.01) and CTR vs. T2 (*p* < 0.05).

## 4. Discussion

In recent years, feed interventions with natural antioxidants have grown because oxidative stress is linked to the development of several diseases in livestock [6,7]. As previously reported, stress can cause oxidative damage of the GI tract with apical mucosal erosion [18]. Moreover, it can cause weaning-induced GI disorders, increase intestinal oxidative stress [19,20], and it is associated with inflammation and post-weaning diarrhea [4]. 

Considering that feed ingredients could affect taste signaling molecules and receptors in the GI tract, our study aimed to test whether dietary high sunflower oil intake with or without verbascoside could affect GI tract morphology and the expression of α-gust and α-tran immunoreactive cells of weaned piglets.

### 4.1. Intestinal Histology and Histometry

No previous study described the GI morphology of piglets under oxidative stress induced by dietary treatment. We focused attention on the small intestine: the duodenum is the first intestinal site in which the microencapsulated verbascoside can affect the intestinal mucosa and in which, consequently, the molecule may display its antioxidant properties [7]. 

Our results showed apical erosion of the villi in the T1 group. However, in the CTR and T2 groups, the micro anatomical structure was quite preserved. At the same time, villi height in the duodenum, jejunum and ileum was similar in the CTR and T2 groups; in T1 group, it was always shorter than in the other two groups. All together, these parameters contribute in evaluating the digestive capacity in piglets and their changes are usually correlated with weight gain and feed conversion ratio [21]. However, in the present study, no differences were observed in weight gain and final weight due to the restrictive regimen adopted, in agreement with the observations of Frankič and Salobir [22] in piglets exposed to n-3 polyunsaturated fatty acids-induced oxidative stress. 

Furthermore, our results showed that in the T1 group, high supplementation of sunflower oil negatively affects villi height and consequently the V:C ratio, but the plant extract containing verbascoside exerts a protective functional role on the morphology of the small intestine in weaned piglets in conditions of oxidative stress. On the same animals, we previously observed that this plant extract modulates oxidative damage in the piglets’ liver without influencing the systemic responses to oxidative stress. Overall, these data demonstrate that the dosage of dietary verbascoside administered in the present study protects the GI tract and liver from oxidative stress induced by a high dosage of sunflower oil, without affecting the systemic response [15]. The use of natural active principles is suggested to enhance growth performances, the immune system, gut morphology and microbiota and to decrease the use of therapeutic antimicrobials. Namkung et al. [23] found no effects on villi height and crypt depth after dietary inclusion of 0.75% natural extracts (cinnamon, thyme and oregano extract). Likewise, other authors did not evidence any differences in villus height and crypt depth in the GI tract of animals fed with 5% carvacrol, 3% cinnamaldehyde, and 2% capsicum oleoresin [24,25]. In contrast, Fang et al. [26] described an increase of the villus height and a decrease of the crypt depth in the small intestine in response to dietary supplementation of *Acanthopanax senticosus* extracts. These results are not conclusively in view of maintaining gut health. The present data give further support to the hypothesis that natural extract supplementation in post-weaning piglets may stabilize gut structure and functions in oxidative stress conditions.

### 4.2. Mucins Profile

Mucus not only acts as a physical–chemical barrier, an important interface between the lumen contents and the intestinal epithelium, but also as a stabilizer of the GI tract and, when digested, as an energy source for microflora [27]. Moreover, in healthy animals, mucus helps to avoid pathogens in the gut lumen invading the mucosa [28]. 

In the present study, the mucin profile was predominantly acidic in the intestinal goblet cells of the T1 and T2 groups, as well as in the Brunner’s glands of the duodenum. However, in the CTR group, the mucin profile was neutral as well as acid. Moreover, the mucus layer was higher in the T1 group than in the CTR and T2 groups. These data suggest that the dosage of dietary verbascoside administered in the present study partially protects the mucosa from oxidative stress induced by a high-fat diet; a correlation between a thick mucus layer and intestinal damage has, in fact, been observed in post-weaning piglets [29]. The thicker mucus layer in T1 animals may protect mucosa from oxidative damage, but may limit the diffusion of nutrients to the apical surface of the epithelial cells, thereby reducing absorption. So, the thinner mucus layer observed in the CTR and T2 groups may contribute to a better feed efficiency in piglets with *ad libitum* feed consumption as previously observed [12].

### 4.3. Expression of Taste Receptors’ Related Proteins

Chemosensing in the GI tract involves G protein-coupled receptors sensing bitter or sweet and umami tastes. They mediate gustatory signaling by interacting with specific subunits, including α-gust and α-tran. Alfa-gust and α-tran immunoreactivities have been localized in the endocrine cells of the stomach and intestine of rodents [30,31], pigs [32], and humans [33], including ghrelin, somatostatin, cholecystokinin, glucagon-like peptide-1 and peptide YY positive cells [13,32].

Bitter taste receptors are important for GI tract defense generated by toxins or plant alkaloids that induce nausea and vomiting or impair gastric emptying [34]. Sweet taste is associated with energetic molecules and might stimulate saliva and gastric and pancreatic secretion to prepare the GI tract for digestion and absorption [30]. In addition, taste receptor agonists produce a secretory response in enteroendocrine cells and induce neuronal activation. They might also be involved in the control of feed intake through gut—brain neural pathways [35].

Dietary treatments did not influence α-gust expression in the GI tract. Previous studies in pigs reported that α-gust expression in pyloric mucosa and the jejunum is disturbed by the short-term and long-term supplementation of a diet containing 35% protein [14]. Otherwise, the expression of α-tran in the duodenum was lower in the T1 group than in the other groups. Other studies reported that the G-proteins, α-gust or α-tran, are signaling molecules that transduce TAS1Rs and TAS2Rs function [13]. 

Our data suggest that the induced oxidative stress affects gut taste receptor function and dietary verbascoside is able to preserve the expression of α-tran in the duodenum, which is the first site in which protected verbascoside can preserve the intestinal mucosa due to its antioxidant property. Considering this, a gradual release in the active principle could be commercially performed in the encapsulation procedure to further reach the intestinal tracts and ensure more extended antioxidant activity. 

The colocalization and the cellular counts of α-tran with chromogranin A and ghrelin were also performed and revealed a partial colocalization with both chromogranin A and ghrelin. Moreover, the percentage of colocalization revealed that the T1 group presented lower cells compared to the CTR and T2 groups. Overall, these data suggest that dietary verbascoside is able to exert a protective role on endocrine cells expressing ghrelin, a hormone involved in many biological processes, ranging from appetite and satiety regulation and growth hormone release, that regulate gastrointestinal motility and secretion processes [36,37]. 

## 5. Conclusions

The present data indicate that dietary supplementation with plant extract titrated in verbascoside is able to preserve gut morphology from induced oxidative stress in weaned piglets. Intestinal morphology revealed that high dosages of sunflower oil (T1) caused detrimental effects on the gut wall of the small intestine and the inclusion of verbascoside is able to protect it from oxidative damage. This has also been confirmed by the observation that the mucus layer was thicker in animals fed the T1 diet. The expression of α-gust was unaffected by dietary treatments. However, the expression of α-tran in the duodenum and the percentage of cells that co-localize α-trans with chromogranin A and ghrelin were lower in the T1 group than in the CTR and T2 groups, suggesting that dietary verbascoside had a protective role by preserving endocrine cells expressing ghrelin.

Overall, these results improve knowledge about sensory perception in piglets and enhance our understanding of the role of feed ingredients, such as fat and polyphenols, in signaling molecules that transduce the G protein subunits α-tran and α-gust involved in gustatory signal transduction in the gut.

## Figures and Tables

**Table 1:** Histometrical analyses of the small intestine in piglets exposed to oxidative stress.

	Dietary Treatment ^2^		
Item ^1^	CTR	T1	T2
Duodenum			
Villi height (µm)	308.70 ^A^ ± 5.78	232.56 ^B^ ± 7.92	290.71 ^A^ ± 1.61
Crypts depth (µm)	253.24 ± 4.13	251.87 ± 1.98	241.39 ± 5.53
V:C ratio	1.21 ^A^ ± 0.01	0.92 ^B^± 0.03	1.22 ^A^ ± 0.02
Jejunum			
Villi height (µm)	278.32 ^A^ ± 5.14	197.53 ^B^ ± 4.14	289.69 ^A^ ± 6.36
Crypts depth (µm)	220.07 ± 1.83	240.92 ± 5.06	254.69 ± 4.52
V:C ratio	1.26 ^A^ ± 0.02	0.83 ^B^ ± 0.03	1.11 ^A^ ± 0.01
Ileum			
Villi height (µm)	340.51 ^A^ ± 6.22	264.60 ^B^ ± 1.67	275.09 ^B^ ± 5.42
Crypts depth (µm)	265.92 ^a^ ± 3.21	229.30 ^b^ ± 3.81	252.48 ^ab^ ± 3.29
V:C ratio	1.29 ^A^ ± 0.04	1.48 ^B^ ± 0.02	1.05 ^C^ ± 0.01

^1^ Values are means ± SEM, *n* = 8; ^2^ CTR—control diet; T1—sunflower oil supplemented diet; T2—sunflower oil supplemented diet plus verbascoside; ^A, B, C^ Means with different superscripts differ significantly (*p* < 0.01); ^a, b,^ Means with different superscripts differ significantly (*p* < 0.05).
